# Reconstruction of the Neopulmonary Root After Coronary Button Harvest for Arterial Switch Operation Using 2-ply Extracellular Matrix (Tyke): A Post-Implant Histology

**DOI:** 10.3389/fcvm.2020.562136

**Published:** 2020-10-30

**Authors:** Steve Bibevski, Mark Ruzmetov, Elena Ladich, Laura E. Mendoza, Frank G. Scholl

**Affiliations:** ^1^Division of Pediatric Cardiothoracic Surgery, The Heart Institute, Joe DiMaggio Children's Hospital, Hollywood, FL, United States; ^2^Section of Pathology, The Heart Institute, Joe DiMaggio Children's Hospital, Hollywood, FL, United States

**Keywords:** extra cellular matrix, ECM, heart repair, histology, pulmonary artery, arterial switch operation, CorMatrix

## Abstract

In children with Transposition of the Great Arteries (TGA), the pulmonary artery, and aorta are connected to the heart abnormally resulting in blue blood (deoxygenated) recirculating to the body and red blood (oxygenated) recirculating to the lungs. The arterial switch operation (ASO) is the standard of care for transposition of the great arteries (TGA), and given the low risk of early mortality and satisfactory long-term outcomes, focus is now on managing longer term complications such as neo-aortic root dilatation, and pulmonary artery stenosis. Since May 2016, we have used 2-ply extracellular matrix (ECM; Tyke) for reconstruction of the coronary button defects using a pantaloon patch. We present histology of implanted 2-ply ECM (Tyke) from a patient who went back to surgery for development of subaortic stenosis ~12 months after ASO.

In a normal heart, there are two large arteries that carry blood out of the heart, the pulmonary artery which carries blood to the lungs, and the aorta which carries blood to the body. In children with Transposition of the Great Arteries (TGA), these arteries are connected to the heart abnormally resulting in blue blood (deoxygenated) recirculating to the body and red blood (oxygenated) recirculating to the lungs. This happens because the aorta is attached to the right-sided pumping chamber instead of the left, and the pulmonary artery is attached to the left-sided pumping chamber instead of the right.

The arterial switch operation (ASO) is the standard of care for transposition of the great arteries (TGA). The arterial switch operation involves cutting off the aorta and pulmonary arteries just above the point where they leave the heart, and reconnecting them to the proper ventricle. The valve stays attached to the ventricle, so what was once the pulmonary valve is now the aortic valve and vice versa. Since the coronary arteries must stay with the aorta, they must be taken off the area above the valve and reimplanted separately above the new aortic valve. This leaves a defect in the vessel from where they were removed, and this requires patching the defect with some type of material (Figure 1).

**Figure 1:**
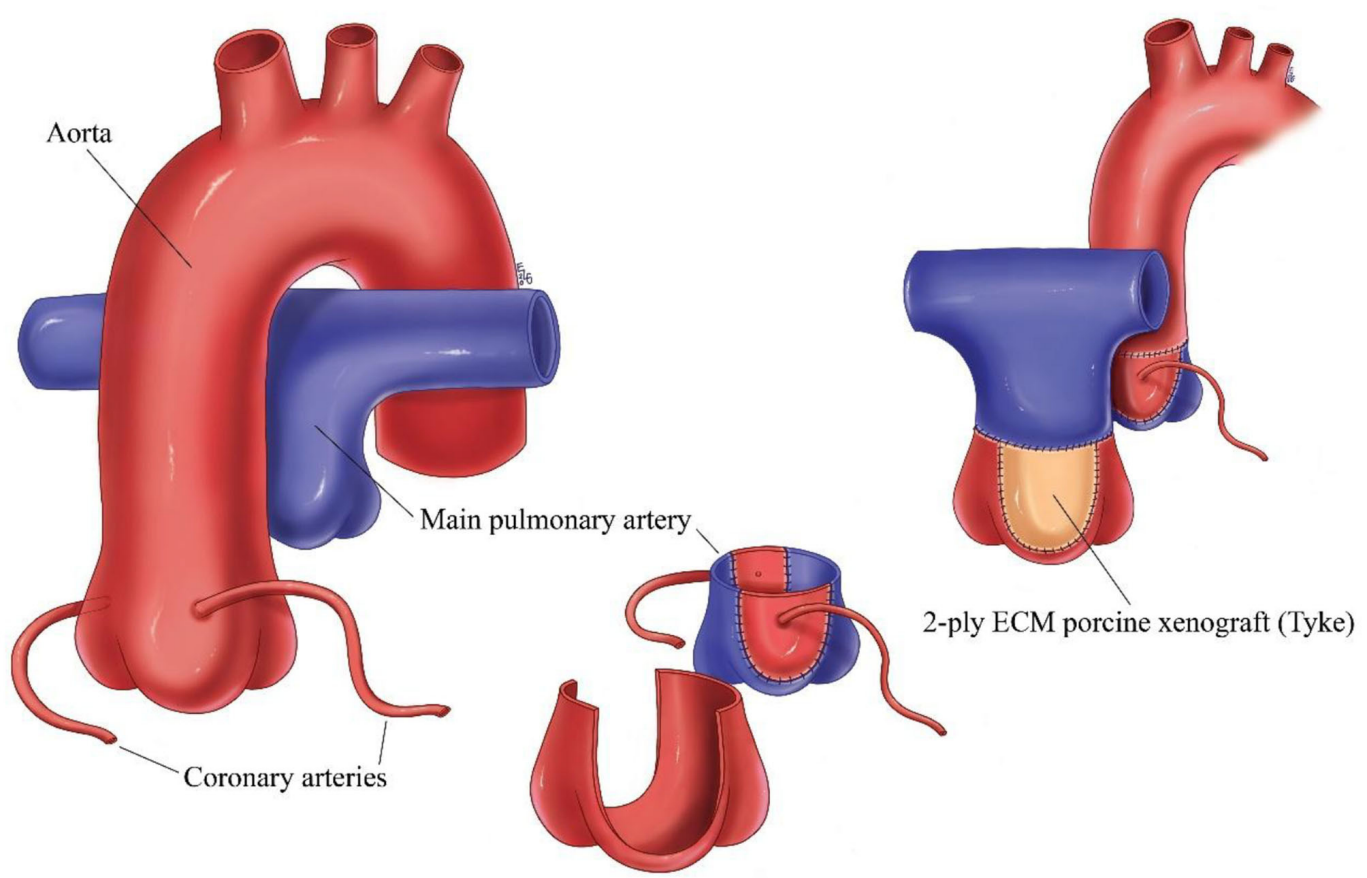
Schematic of arterial switch operation demonstrating the translocation of the great vessels, removal of the coronary arteries and patching of the defect left behind from removal of the coronary arteries with ECM.

Given the low risk of early mortality and satisfactory long-term outcomes, focus is now on managing longer term complications such as neo-aortic root dilatation, and pulmonary artery stenosis (1). The incidence of pulmonary artery stenosis following ASO is surprisingly relatively high (2). Although relief with intervention is generally effective, technique used for reconstruction of the coronary buttons and material used may impact on the incidence. Since May 2016, we have used 2-ply extracellular matrix (ECM; Tyke) for reconstruction of the coronary button defects using a pantaloon patch. We present histology of implanted 2-ply ECM (Tyke) from a patient who went back to surgery for development of subaortic stenosis ~12 months after ASO.

## Clinical Summary

The original surgery was performed in a patient with prenatal diagnosis born at 37 weeks with no intrapartum complications. Postnatal echocardiographic diagnosis of double outlet right ventricle with TGA (aorta from right ventricle, anterior and right of pulmonary artery), sub pulmonic ventricular septal defect (VSD) with overriding pulmonary artery was made. Patient underwent repair at 11 days old where an arterial switch procedure and closure of VSD was performed. A LeCompte maneuver was performed and the posterior neo-pulmonary artery root where the coronary buttons were harvested was reconstructed using a pantaloon shaped patch of 2-ply ECM core porcine xenograft (Tyke) with running 7-0 Prolene suture. The patient did well and was discharged at 18 days postoperatively.

Post-operatively the patient was evaluated by the genetics service and a diagnosis of Sotos syndrome (chromosome disorder 5q35.2q35.3 duplication that interrupts NSD1 gene) was made which is associated with congenital cardiac defects. At 11 months of age, a follow up echocardiogram revealed significant sub-aortic stenosis. The patient was referred for cardiac catheterization which revealed significant outflow tract gradient of 92 mmHg and the patient was referred for surgical repair.

During this procedure, the aorta and pulmonary arteries were separated easily. The pulmonary artery was transected allowing access to the aorta which was also transected. This allowed for inspection of the left ventricular outflow tract through the aortic valve which revealed an exceedingly tight opening in the left ventricular outflow tract measuring ~3 mm in diameter. A longitudinal incision was carefully made down into the left ventricle and connected with a transverse incision excising a large chunk of septal left ventricular outflow tract muscle, now allowing a 10 mm dilator to pass easily. Before re-anastomosing the pulmonary artery, the pantaloon patch was examined from the inside and could not be distinguished from the native tissue except for the suture line. The tissue looked and felt like normal pulmonary artery. A small 1 mm sample of the pulmonary artery anastomosis was therefore taken from the rim of pantaloon patch for histology to evaluate the destiny of the inserted ECM patch. The patient recovered well, without any complications, and was discharged uneventfully.

The specimen was dehydrated in a graded series of ethanol and infiltrated with paraffin. The block was sectioned at 4–6 microns, mounted on a glass slide, and stained with hematoxylin and eosin and Masson's trichrome stains. In addition, immunohistochemistry for CD31 was performed on the paraffin block.

**Figure 2:**
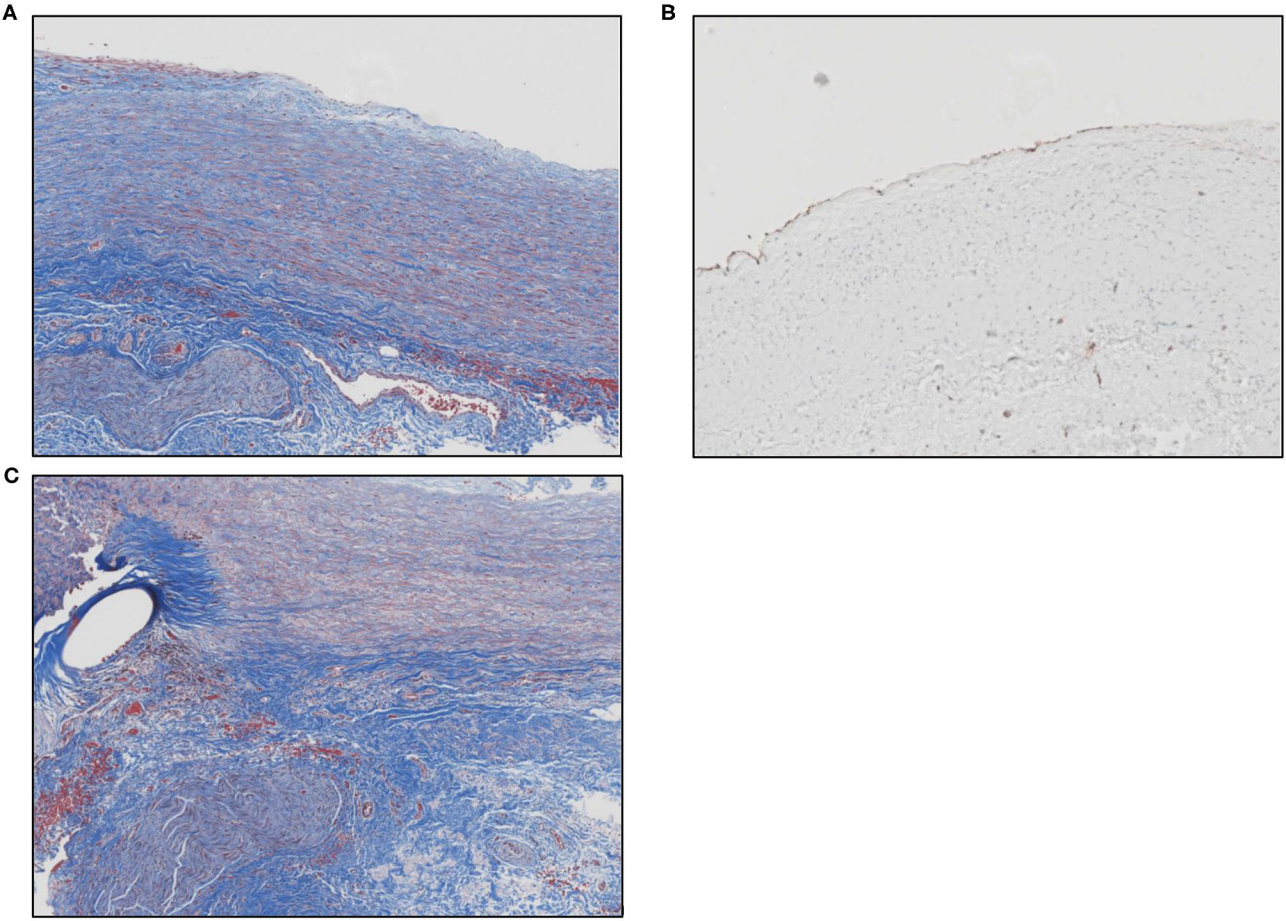
**(A)** Masson's trichrome stain shows the remodeled ECM scaffold material. The neo-tissue is characterized by a highly aligned and organized accumulation of spindle cells and collagen fibers oriented along the length of the pulmonary artery. The luminal surface (top) is lined by endothelial cells and there is a vascular adventitia on the abluminal surface. Sections of a nerve fiber can also be seen at the lower left corner of the image. **(B)** Image showing positive immunolabeling for CD31+ endothelial cells along the luminal surface of the remodeled graft material. **(C)** Masson's trichrome stain shows the anastomotic site between the native pulmonary artery and the graft material. The oval unstained space at the left represents the site of suture placement. The graft material (to the right of the suture) is characterized by an organized, aligned accumulation of collagen fibers and spindle-shaped cells. The bottom portion of the picture shows the vascular adventitia with a section of nerve fiber.

Microscopic evaluation of the graft at the pulmonary anastomosis showed a small region of acellular laminated Cormatrix collagen with surrounding spindle cells and collagen deposition with re-endothelialization of the surface demonstrated by CD31 staining. Overall, inflammation was mild and consisted predominantly of lymphohistiocytic inflammation around suture material, nerve fiber, and at the interface with the ECM patch material. Figure 2 shows the histology of remodeled ECM scaffold material.

**Figure 3:**
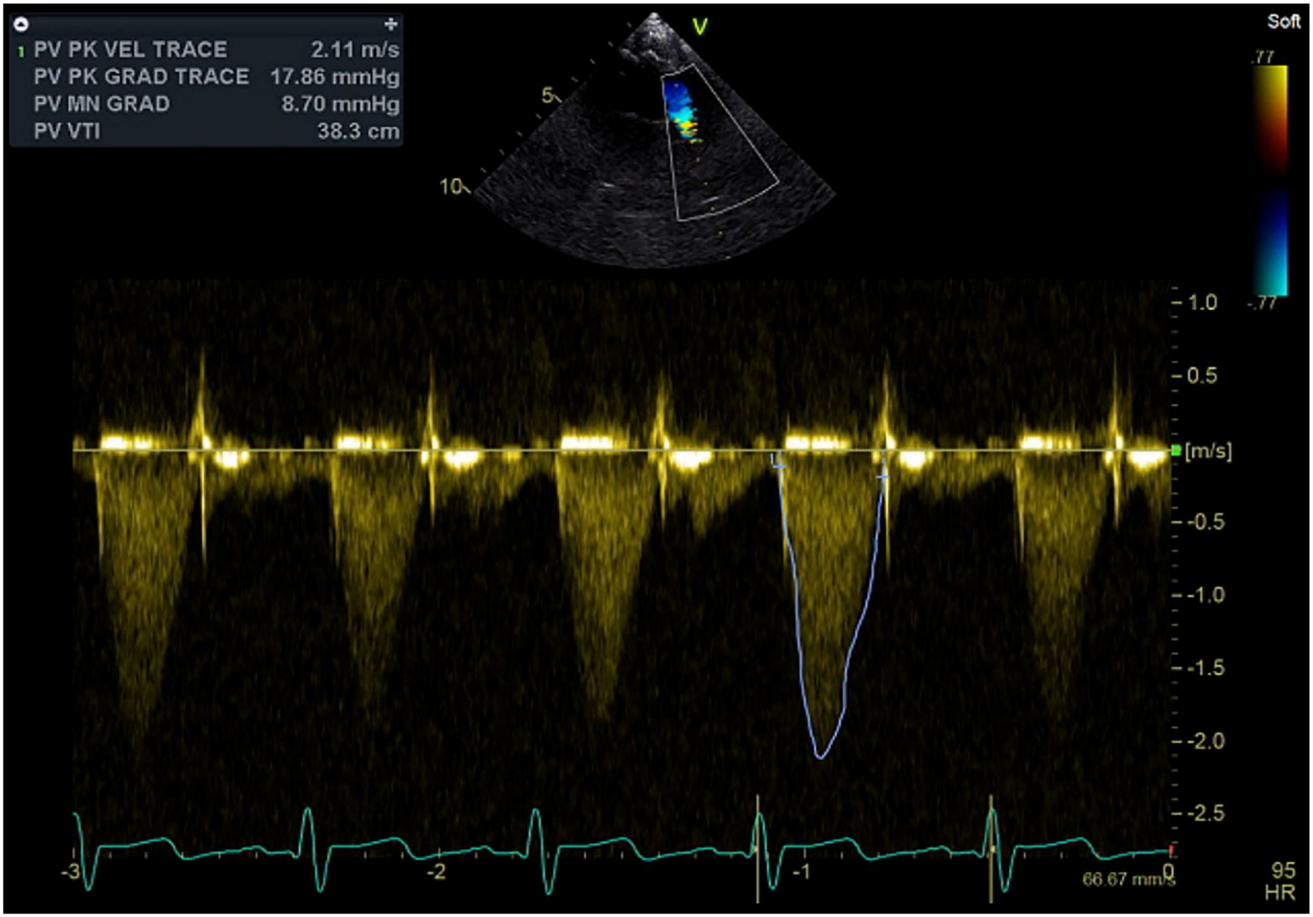
Continuous wave Doppler signal with the sample volume at the level of the pulmonary outflow tract, neo-pulmonary valve, and pulmonary trunk demonstrating well-functioning valve and absence of obstruction.

Postoperative echocardiography performed more than year and half after initial switch operation demonstrated nicely functioning pulmonary valve with trivial pulmonary insufficiency and 18 mmHg peak pulmonary gradient (Figure 3).

## Discussion

Decellularized matrices are biological grafts capable of stimulating *in vivo* migration and proliferation of endothelial cells, recruitment and differentiation of mural cells, culminating in the formation of a bio integrated tissue (3). CorMatrix ECM, is an extracellular matrix (ECM) derived from porcine small intestinal submucosa. It offers potential for natural in growth and development of native arterial cells and structure (3, 4). Cormatrix ECM has been used for repair of a variety of cardiac structures including valvular, arterial, and pericardial sites. In several studies, use of CorMatrix for valve repair has been associated with an intense inflammatory response in the surrounding native tissue, the typical response included macrophages and giant cells in contact with the material, surrounded by lymphocytes, macrophages, plasma cells, and eosinophils; and little or no remodeling to form tissue resembling a 3-layered native valve was seen at ≤9 months after implantation (4, 5). However, in another preliminary experience with cardiac reconstruction using decellularized porcine ECM scaffold, the explanted tissue showed resorption of the SIS-ECM, replacement with organized collagen, and re-endothelialization (6). Longer-term follow-up was suggested to assess the potential for growth. A recent pathology study evaluating Cormatrix tissues in a variety of cardiovascular surgical sites demonstrated that the Cormatrix tissue itself remained acellular up to 2 years post-implantation but appeared to serve as a scaffold onto which new tissue formed, akin to neointimal proliferation (7). Neointimal thickness increased over time and a limited amount of elastic fiber deposition was observed at timepoints beyond 2 years. Interestingly, severe chronic inflammation was an uncommon finding in arterial repairs, including anastomotic sites. The authors postulated that the degree of inflammatory response observed in explanted Cormatrix tissue may be dependent on anatomic site with great vessel grafts less prone to inflammation as compared to valvular grafts (7). In our patient's arterial scaffold, mild chronic inflammation was observed, in keeping with these observations. A variety of other factors may explain the variation in inflammation severity observed between studies including variable immunologic responses to the porcine tissues, patient age, and clinical setting, e.g., graft failures removed as surgical specimens or at autopsy.

Bioscaffolds such as Cormatrix become incorporated by host tissue cells with eventual collagen deposition characteristic of wound healing (8). There have been no reports to date proving *de novo* growth of true histologic three layered arteries or valves resembling native cardiac structures in human explants. At ~1 year post-implantation in a pediatric patient, we report incorporation of the Cormatrix scaffold by collagen deposition, re-endothelialization, and mild chronic inflammation, reflecting a typical healing response for implant duration similar to other reports. Additional studies evaluating longer term explants may further elucidate the mechanisms of tissue incorporation and potential for reconstitution of native structures.

This research did not receive any specific grant from funding agencies in the public, commercial, or not-for-profit sectors.

## Data Availability Statement

The raw data supporting the conclusions of this article will be made available by the authors, without undue reservation.

## Ethics Statement

The studies involving human participants were reviewed and approved by Memorial Healthcare System Office of Human Research. Written informed consent to participate in this study was provided by the participants' legal guardian/next of kin.

## Author Contributions

All authors listed have made a substantial, direct and intellectual contribution to the work, and approved it for publication.

## Conflict of Interest

The authors declare that the research was conducted in the absence of any commercial or financial relationships that could be construed as a potential conflict of interest.
